# Proper glove use: a multi-centre before–after improvement project

**DOI:** 10.1016/j.infpip.2026.100574

**Published:** 2026-08-07

**Authors:** Anne F. Voor in ’t holt, Robbert G. Bentvelsen, Fleur Adriaanse, Sabrina K. Been, Vicki Erasmus, Juliëtte A. Severin, the Proper Glove Use project group, Jojanneke Dekkers, Tineke Heijerman, Yara Bamberg, Greet van der Tempel, Marianne van Groningen, Pia Gutierrez Zarate, Mieke Waltmans-den Breejen, Joy Kersten, Henriette Bakker, Bas de Jong, Wim van Vianen, Evelien van der Linden, Anja Bakker

**Affiliations:** e Adrz, the Netherlands; f Albert Schweitzer ziekenhuis, the Netherlands; g Argos Zorggroep, the Netherlands; h Curamare, the Netherlands; i Erasmus MC, the Netherlands; j Franciscus Gasthuis & Vlietland, the Netherlands; k Het Spectrum - Salios, the Netherlands; l Ikazia ziekenhuis, the Netherlands; m Rijndam Revalidatie, the Netherlands; n Zorgorganisatie de Blije Borgh, the Netherlands; o ZorgSaam Zorggroep, the Netherlands; a Department of Medical Microbiology and Infectious Diseases, Erasmus MC University Medical Center, Rotterdam, the Netherlands; b Infection Prevention and Antimicrobial Resistance Care Network South-Western Netherlands, Rotterdam, the Netherlands; c Department of Public Health, Erasmus MC University Medical Center, Rotterdam, the Netherlands; d Microvida Laboratory for Microbiology and Immunology, ZorgSaam Ziekenhuis, Terneuzen, the Netherlands

**Keywords:** Gloves, Protective, Infection control, Behaviour, Sustainability

## Abstract

**Introduction:**

In healthcare, excessive glove use increases the risk of spreading micro-organisms and amount of plastic waste. With no sustainable alternatives, reducing glove use is the primary mitigation strategy. The aim of the project was to understand healthcare workers’ (HCW) perspectives on glove use, to evaluate if a behaviour change was achieved through an intervention, and to measure the reduction in glove use.

**Methods:**

A multi-centre before–after improvement project was conducted in the south-western region of the Netherlands. First, a regional policy on glove use was formulated. At baseline, a Capability, Opportunity, Motivation – Behaviour (COM-B) questionnaire on glove use was distributed among HCW. The intervention comprised a toolbox for healthcare facilities and observations of glove use. Additionally, glove procurement statistics were gathered, and interviews about the perceived usefulness of the intervention were conducted.

**Results:**

The questionnaire had 1111 participants. The COM-B theme Opportunity scored lowest, while Behaviour scored highest. At baseline, 1550 observations were performed by 15 healthcare facilities, postintervention 940 observations were performed by six healthcare facilities, showing a significant decrease in unnecessary glove use by HCW (42.3% vs 25.9%, *P* < 0.001). Glove procurement statistics showed a significant decrease in one healthcare facility (*P* < 0.001). The overall grade of the intervention given by HCW was a 7.8 out of 10.

**Conclusion:**

This project highlights the need for targeted interventions to improve issues related to the opportunity to wear gloves correctly. While the percentage of unnecessary use was notably high, interventions chosen by HCW can reduce this significantly, with potentially a positive impact on patient care and the environment.

## Introduction

Billions of gloves are used annually across the globe for occupational health and safety and infection prevention and control (IPC) in healthcare. They are essential as personal protective equipment (PPE) when anticipating contact with blood or other infectious materials, mucous membranes, non-intact skin, potentially contaminated skin, contaminated equipment, and harmful substances, or needed as a transmission-based precaution [1]. However, inappropriate or incorrect glove use can have unintended consequences, including the spread of micro-organisms [2]. Another consequence is the contribution to hospital waste and global warming. A study by Hunfeld *et al.* showed that the highest carbon footprint was caused by disposable gloves, syringes, and disposable medical clothing, with gloves being identified as a key ‘environmental hotspot’ that requires urgent intervention and has opportunities for circular innovation [3]. Disposable gloves currently lack sustainable alternatives, making the reduction of their use the primary strategy to mitigate these risks.

In 2020, the coronavirus disease 2019 (COVID-19) pandemic brought a new situation with continuous challenges of shortages of PPE. Glove shortages led to the awareness that gloves should only be used when absolutely necessary, ensuring their availability for COVID-19 patient care to protect healthcare workers (HCW). This awareness emerged together with the awareness of the negative environmental impact of these large amounts of disposables. As HCW protection was felt as a first priority, prolonged or extended glove use was sometimes practised in settings where COVID-19 patients were cared for in large cohorts, increasing the risk of transmission of pathogens from patient to patient [4]. In 2021, the Infection Prevention and Antimicrobial Resistance Care Network South-Western Netherlands (IPCN-SWN) identified proper glove use as an IPC topic to focus on, for which a project was developed and carried out.

In this report, we describe the steps that were taken in this project, and its effects. It involved the inventory of views of HCW on their use and misuse of gloves and an estimation of its relationship with observed behaviour. Based on these, a toolkit was developed for the participating healthcare facilities as an intervention. We measured if a behaviour change was achieved and if the overall use of gloves was changed by the introduction of the intervention in a before–after design with a control group. Lastly, the perceived usefulness of the intervention was investigated.

## Methods

### Study design

This multi-centre before–after project with intervention and questionnaires was designed for institutional healthcare improvement. In Dutch, this project is called ‘Goed Gebruik Handschoenen’ (GGH), meaning ‘Proper Use of Gloves,’ and it was initiated by the IPCN-SWN [5,6]. The IPCN-SWN includes healthcare facilities (i.e. hospitals and long-term care facilities [LTCFs]) in the Rotterdam Rijnmond, Zuid-Holland Zuid, and Zeeland regions of the Netherlands, which together have > 2 million inhabitants. This project was conducted between 2021 and 2023. A timeline of the project is available in Additional figure 1. Contact persons from all participating centres gave written approval to publish the anonymized results of this project. This project followed the Standards for Quality Improvement Reporting Excellence guidelines [7].

### Project kick-off

On 5^th^ March 2021, the IPCN-SWN organized an online 1.5-h project kick-off meeting, which was promoted through e-mail newsletters, leaflets, and social media. In this meeting, the GGH project was introduced, including scientific background on the topic, and a discussion was held to identify healthcare facilities’ needs to raise awareness about proper glove use. HCW (i.e. primarily IPC practitioners) of healthcare facilities could subsequently express interest in participating in the GGH project. Additionally, a brief introduction to the project and an invitation to participate were sent via relevant e-mail lists and shared via social media, newsletters, and during meetings of other projects within the IPCN-SWN.

### Participating centres

After the kick-off meeting and other recruitment efforts, healthcare facilities could formally sign participation commitment forms. By signing a participation commitment form, the idea was that contact persons from healthcare facilities were more likely to feel a personal sense of responsibility and intrinsic motivation to follow through with their involvement in the project. Each participating healthcare facility had to appoint a local project leader who took part in project meetings. Furthermore, the local project leader had the option to participate in the project advisory board. The project advisory board consisted of 13 people from diverse fields of expertise, including clinical microbiologists, IPC practitioners, a communication advisor, a behavioural scientist, the programme manager and project manager of the IPCN-SWN, and an advisor for LTCFs. The role of the advisory board was to give feedback on the GGH project’s activities and progress. The advisory board met four times in the period from May 2021 to January 2023. After that, the advisory board communicated via e-mail.

In November 2021 and March 2022, two local project leader meetings were organized. During the first meeting in November 2021, the explanation of the developed toolkit and its implementation process were central topics. The second meeting in March 2022 was dedicated to evaluating the GGH project. Additionally, in November 2021, an online meeting was held to address remaining questions about the project. This meeting was recorded and published on YouTube, allowing participants who were unable to attend to watch it later [8]. Because different policies and protocols between the participating healthcare facilities on the use of gloves were in effect, a key step in this project was to come to an agreement on proper glove use and develop a regional policy. This regional policy was developed separately for hospitals and for LTCFs, and is available in Dutch online [9,10].

### The questionnaire

To gain insight into the knowledge of HCW regarding the use of gloves, the existing Capability, Opportunity, Motivation - Behaviour (COM-B) questionnaire that had been used to study hand hygiene was adapted to focus on glove use [11,12]. The questionnaire was translated from English into Dutch by van Dijk *et al.*; the GGH project team replaced all references to hand hygiene by gloves, and the theme Behaviour was adjusted to reflect the developed regional policy (Additional file 1, Additional file 2).

### The intervention – toolkit and observations

#### The toolkit

Based on the input received during the GGH kick-off meeting and in cooperation with the local project leaders, tools for the toolkit were designed (Additional file 2). The final developed box was sent to participating centres with all printable materials in it starting from 16^th^ November 2021. All available tools were published on the project website [13]. In March 2022, the toolkit was evaluated in the local project leaders’ meeting. During this meeting, it became clear that an additional poster was needed, with pictures showing how to safely put on and take off gloves. Additionally, it was expressed that it would be beneficial to develop stickers with reminders of proper glove use. Both items were subsequently developed and included in the toolkit and made available online.

#### Observations

Local project leaders had the option to only choose tools from the toolkit or to also measure proper glove use via observation tools (Additional file 2). This was not mandatory, but strongly recommended. When performing measurements, it was advised to first do a baseline-before measurement, followed by implementing the toolkit, and finally a second-after measurement. It was also possible to only do a baseline measurement without further follow-up measurements. A manual on how to perform the observations on proper glove use was created together with a communication advisor and send to the local project leaders. A Google Form was developed for the glove observations. It was recommended to collect data from the same departments when conducting before-and-after measurements to facilitate analysis and reporting. After the ‘before’ measurements were completed, the GGH project team created reports per observed department. It was advised to communicate the results with the respective department and to choose and implement tools from the toolkit together. For departments also performing ‘after’ measurements, a final report was created showing the differences. Departments from two hospitals outside of the IPCN-SWN were asked to be the control group. They did not have access to the toolkit and only did two rounds of observations with some months in between.

### Glove procurement figures

To measure if the overall use of gloves was changed by the introduction of the intervention, local project leaders were asked to provide monthly glove procurement figures starting from 2019 until 2023 for each included department.

### Perceived usefulness

To assess the perceived usefulness of the provided toolkit and observation tools provided by the GGH project team to the participating healthcare facilities, interviews with local project leaders were conducted. The interview questions were developed by F. A., V. E., R. B., and S. B. (Additional file 3). Between July and November 2023, all local project leaders were called and interviewed over phone by F. A. and by a student. Answers to predefined questions were noted in a Microsoft Excel document.

### Statistical analysis

Before analysis, the data were cleaned. Since text fields were used in the COM-B questionnaire, some respondents left questions unanswered; these were recorded as missing data. The theme percentage was calculated by dividing the score of a subscale by the maximum score that could be achieved for that subscale. The maximum score was calculated by multiplying the number of respondents of the questionnaire by 30 for Capability (i.e. 6 questions, maximum of 5 points), by 25 for Opportunity and Motivation (i.e. 5 questions, maximum of 5 points), and by 90 for Behaviour (i.e. 18 questions, maximum of 5 points). Descriptive analyses were performed, calculating percentages, median, and means, when appropriate. Proportions between groups were compared using the *χ*^2^ test. A value of *P* < 0.05 was considered statistically significant. For the analyses, IBM SPSS statistics version 28.0.1.0 (build 142) was used (IBM Corp., Armonk, New York, USA).

## Results

### Inclusion

The GGH kick-off meeting in March 2021 resulted in 65 applications from 26 individual healthcare facilities. Eventually, 23 healthcare facilities participated in this project. All participating healthcare facilities were invited to fill out the modified COM-B questionnaire and invited to provide glove procurement figures. Twelve out of 23 facilities used the observation tools to observe glove use and misuse. The other 11 participants did only use items from the toolkit.

### The modified COM-B questionnaire

Between December 2021 and June 2022, 1111 HCW respondents from 13 different healthcare facilities completed the modified COM-B questionnaire. Two hundred and ninety-two HCW were from LTCFs (26.3%) and 819 from hospitals (73.7%), including one rehabilitation clinic. Regarding HCW function, 397 different functions were reported, which were categorized in 26 different categories. More than half were nurses (*N* = 583, 52.5%), followed by practical nurses (*N* = 132, 11.9%), nurses in training (*N* = 62, 5.6%), laboratory technicians (*N* = 57, 5.1%), and nursing assistants (*N* = 48, 4.3%). The median age of the 1111 HCWs was 40, ranging from 16 to 68. The COM-B theme Opportunity scored lowest and Behaviour scored highest (Additional file 4). This did not differ between hospitals and LTCFs (Additional file 4). Regarding HCW function, nursing assistants scored highest on all four themes (Additional file 4).

### Observations

#### The intervention group

The 12 participating healthcare facilities performed glove observations between December 2021 and March 2023. Baseline/before measurements took place between December 2021 and September 2022, and after measurements between April 2022 and March 2023. The number of participating hospitals and LTCFs was lower in the after period (Table I). Nurses were mostly observed (Table I). The number of HCW who were not wearing gloves when they should according to policy significantly decreased following the intervention (7.7% vs 4.7%, *P*-value = 0.045, Table I). Similarly, the number of HCW not wearing gloves when policy indicated that they should not significantly increased (57.7%–74.1%, *P* < 0.001), with unnecessary glove use decreasing from 42.3% to 25.9% (Table I). Various situations where HCW were using or wearing gloves incorrectly were recorded. A summary is available in Additional file 6 Tables I and II, with no hand hygiene before putting on gloves being the most frequently reported issue both in the before and after periods (26.1% and 32.1%, respectively), followed by no hand hygiene after taking off gloves also in both periods (12.7% and 16.9%, respectively).

**Table I:** Before–after glove observations

	Intervention group		Control group	
	Before (%)	After (%)	Before (%)	After (%)
No. of participating hospitals	10	5	2	2
No. of participating departments	56	30	11^a^	10^a^
No. of participating LTCFs	5	2	0	0
No. of participating departments	17	2	0	0
Healthcare workers^b^	1550	1044	293	272
Nurse	958 (61.8)	781 (74.8)	215 (73.4)	211 (77.6)
Practical nurse	129 (8.3)	23 (2.2)	0 (0)	0 (0)
Hospital cleaner	87 (5.6)	30 (2.9)	33 (11.3)	21 (7.7)
Laboratory technician	87 (5.6)	84 (8.1)	0 (0)	0 (0)
Dietary aide	83 (5.4)	64 (6.1)	13 (4.4)	14 (5.1)
Physician	68 (4.4)	32 (3.1)	8 (2.7)	11 (4.0)
Nursing assistant	57 (3.7)	17 (1.6)	7 (2.4)	6 (2.2)
Doctor’s assistant	30 (1.9)	0 (0)	0 (0)	0 (0)
Nurse in training	16 (1.0)	0 (0)	0 (0)	0 (0)
Other	35 (2.3)	13 (1.2)	17 (5.8)	9 (3.3)
Total no. of observations	1550	1042^c^	293	272
HCW should wear gloves	818	425	131	136
HCW wears gloves (%)	755 (92.3)	405 (95.3)	120 (91.6)	123 (90.4)
HCW does not wear gloves (%)^e^	63 (7.7)^e^	20 (4.7)^e^	11 (8.4)	13 (9.6)
HCW should not wear gloves	732	617	162	136
HCW wears gloves (%)	310 (42.3)	160 (25.9)	68 (42.0)^f^	32 (23.5)^f^
HCW does not wear gloves (%)	422 (57.7)^f^	457 (74.1)^f^	94 (58.0)	104 (76.5)
HCW wears gloves	1057	561^d^	188	155
Correct (%)	409 (38.7)	231 (41.2)	71 (37.8)	62 (40.0)
Incorrect (%)	648 (61.3)	330 (58.8)	117 (62.2)	93 (60.0)

No., number; LTCFs, long-term care facilities; HCW, healthcare worker.

a Including one category indicated as ‘other.’

b > 15 for each HCW function in the intervention or the control group, otherwise indicated as ‘other’.

c For 2 observations, it was not noted if the HCW should wear gloves, and these 2 observations were indicated as missing.

d For six HCW, information about correct use was missing.

e Statistically significant difference, *P*-value = 0.045.

f Statistically significant difference, *P*-value < 0.001.

**Table II:** Perceived usefulness of 11 participating healthcare facilities performing glove observations

Interview question	Hospitals *N* = 7^b^	LTCFs *N* = 4
Which items from the toolkit were used (%)?		
Facts and myths	6 (85.7)	4 (100)
Posters	5 (71.4)	4 (100)
Videos	6 (85.7)	2 (50)
Game	6 (85.7)	2 (50)
Stickers	3 (42.9)	2 (50)
Icons	3^a^ (50)	2 (50)
Image library	2 (28.6)	1 (25)
Was the regional policy adopted? (answer ‘Yes’)	4 (57.1)	3^a^ (100)
What grade do you give the toolkit? (mean and range)	7.9 (7–8.5)	7.7 (7-8)
Which item of the toolbox did you favour the most?	Posters (*N* = 5), videos (*N* = 3), game (*N* = 2), stickers (*N* = 1)	Posters (*N* = 3), videos (*N* = 1)
Which item of the toolbox did you favour the least?	Game (*N* = 2), icons (*N* = 2), facts & myths (*N* = 1), stickers (*N* = 1), videos (*N* = 1)	Game (*N* = 1), stickers (*N* = 1), videos (*N* = 1)
Open questions:		
How was the toolkit distributed/implemented?	Via newsletters, department meetings, lunch meetings, infection committee meetings, via hand hygiene education meetings, shared with a contact person at the departments, and via organized GGH project meetings.	
How could the toolbox be improved?	Make the game more difficult, add a tool to calculate the effect on CO_2_ emission on reduced glove use, add an app to perform glove observations, have gadgets to hand out, and add an item with fluorine to show what happens when you take your gloves off.	
How could the healthcare improvement project be improved?	More attention to perceived usefulness and motivation of HCW to participate, a top-down approach, in addition to the current bottom-up strategy, might be beneficial, more interaction from the project team with the local project leaders could be beneficial as well as more opportunities to interact with other local project leaders, emphasize that dedicated project time is needed, and ensure that only one such project is implemented per department.	

LTCFs, long-term care facilities; *N*, number; GGH, Goed Gebruik Handschoenen project, HCW; healthcare workers.

a One local project leader indicated that the answer was unknown.

b One local project leader indicated that the answers were for both the hospital and LTCF; these answers were categorized as ‘hospitals.’

#### The control groups

Two hospitals not participating in the GGH project and outside of the IPCN-SWN region agreed to be control hospitals and performed glove observations without implementing any GGH interventions. Before-observations took place in January and February 2022, and after-observations in May, June, and July 2022, giving -two to five months’ time in between observations. The two hospitals together performed 293 before- and 272 individual after-observations, mostly observing nurses (> 70%, Table I). Over 90% of HCW in the before and after periods were wearing gloves, when this was indicated (Table I). When wearing gloves, this was incorrect in around 60% of observed moments in both periods (Table I). There were various reasons why HCW were wearing gloves incorrectly. A summary is available in Additional file 6 Table III, with touching surfaces and items in the (patient) environment being the most frequently reported reason in both the before period (42 out of 117, 35.9%) and in the after period (29 out of 93, 31.2%) (Table I). When HCW should not wear gloves, 42% in the before and 23.5% in the after period did wear gloves, which is a significant reduction (*P* < 0.001).

### Glove procurement figures

From seven healthcare facilities, glove procurement figures were obtained, of which six provided numbers per month, which could be used for analyses. When plotting the data, it became clear that procurement statistics from 2020 and 2021 were unstable due to the COVID-19 pandemic (Additional file 5). Therefore, figures from 2019 were compared with figures from 2022, for which the data were available for three healthcare facilities (i.e. two hospitals and one LTCF). In one healthcare facility, a hospital, glove procurement figures were significantly lower in 2022 compared to 2019 (*P* < 0.001) (Additional file 5).

### Perceived usefulness

Eleven out of 12 participating healthcare facilities (91.7%) in which glove observations were performed were interviewed by phone about their perceived usefulness of the intervention. The overall grade of the intervention was 7.8 out of 10. The most frequently used tool from the toolkit was the ‘facts and myths,’ while the image library was used the least. In comparison, posters were the most favoured tool, and the game was the least favoured (Table II). Stickers to place on glove boxes were specifically asked for during our project leader meetings; however, they were only used by half of the participating centres and it was also indicated as both most and least favoured (Table II). Additionally, several points of improvement were raised (Table II).

## Discussion

### Summary of evidence

This project found that the intervention, perceived as useful by HCW, significantly improved proper glove use. The percentage of observed moments that HCW should wear gloves and did not was low and significantly decreased (i.e. increasing safety), and the percentage of observed moments that HCW should not wear gloves and did not significantly increased (i.e. increasing safety and sustainability). In recent years, similar initiatives on proper glove use have been undertaken across the globe. The 2025 World Health Organization (WHO) World Hand Hygiene Day (WHHD) stated as objective to promote times for appropriate glove use and to raise awareness of the environmental and climate impact of gloves, with as slogan ‘It might be gloves. It’s always hand hygiene’ [14]. Other initiatives include: Royal College of Nursing, The United Kingdom, introducing Glove Awareness weeks since 2018 [15], the National Health Service (NHS) Scotland with the slogan ‘Be glove aware to protect our people and our planet’’ [16], and the NHS ‘the gloves are off!’ campaign [17]. In Australia, Stackelroth *et al.* launched the ‘No Risk? No Glove!’ campaign in December 2020, and Wilkie-Miskin *et al.* implemented the ‘Gloves Off!’ intervention in 2023–2024 [[18], [19], [20]]. In Canada, the campaign named – ‘Les gants, pas tout le temps!’ was launched in Quebec in 2023 [21]. Lalakea *et al.* performed a ‘Use gloves wisely’ educational intervention in an outpatient setting in the United States of America. Most of these initiatives have their focus on reducing glove use, while there is also the aspect of correct or proper use, which was included in our project. We identified that the main reason why HCW were using gloves incorrectly, as based on observations, was that no hand hygiene was performed before putting on and after taking off gloves. This is in line with the findings by Kristiansen *et al.*, who performed 73 glove observations in nursing homes, with a lack of hand hygiene after glove removal that was observed in 36 out of 67 instances (53.7%) [22]. This behaviour introduces a risk for both patients and HCW. Another remarkable risk we observed, although being in low numbers, was HCW touching their face with gloves on. It underscores the critical link between hand hygiene and glove use. Teesing *et al.* showed that targeted interventions in nursing homes achieved significant positive changes in hand hygiene with donning and doffing gloves as well as a significant decrease in inappropriately unchanged gloves after contact with body fluids [23]. Hand hygiene interventions may, however, also have unexpected and undesirable effects. Santosaningsih *et al.* showed a reduction in use of gloves when they should not be used, but the use of alcohol-based hand rub on gloves increased significantly among nurses and students [24]. Therefore, future IPC initiatives should integrate both hand hygiene and glove use, as was done in the 2025 WHO WHHD campaign [14]. The challenge will be to sustain HCW engagement with the topic. Continued monitoring and repeated interventions are needed to maintain currently achieved levels, as compliance often declines over time, a pattern well documented in previous hand hygiene initiatives [25]. Many healthcare facilities already use mobile applications to monitor compliance of hand hygiene. Ideally, these should include glove use, as this will facilitate healthcare facilities with the observations, especially the analysis of results so that targeted interventions can be chosen. A toolkit as was used in our project provides the HCW the opportunity to choose a set that matches best with the local needs and culture, which is considered a key element for success in line with the ‘Adapt to Adopt’ slogan from the WHO [26]. As an example, in the analysis of perceived usefulness, the game was considered the most favourite item by some healthcare facilities, while others indicated this item as the least favoured. Feedback was also given to improve the game by including more difficult questions, and this feedback is useful for follow-up initiatives.

A study by van Dijk *et al.* showed that for hand hygiene compliance, higher scores for Opportunity and Motivation were significantly associated with observed hand hygiene compliance on a ward [12]. In our project, Opportunity scored lowest in both hospitals and LTCFs. This indicates that future initiatives should focus on improving opportunities for HCW to wear gloves correctly, alongside improving opportunities for hand hygiene. Practical implications could entail reliable availability of gloves in all required sizes, positioning them at the point of care, standardizing their placement, and streamlining work flows [27]. However, this is complicated. As shown by the studies by Flores *et al.*, Loveday *et al.*, and Kristiansen *et al.*, multi-modal interventions are necessary to improve practice, not only aimed at Opportunity, but also aimed at Motivation, Behaviour, and Capability [22,28,29]. The WHO estimates a shortage of 11–18 million HCW by 2030 [30]. These shortages affect countries across all levels of socioeconomic development and are likely to influence IPC policy compliance, including hand hygiene and gloves use. Reduced staffing levels are known to increase workloads and stress, which in turn limit the time and attention HCW can devote to IPC practices. Additionally, the growing trend of nursing self-employment in many countries may further affect knowledge, motivation, and compliance to hospital’s IPC and glove policies [31].

A remarkable finding in our project was that the control hospitals significantly improved, with similar levels of incorrectly worn gloves in the before and after observation. We hypothesize that due to the attention the project received on social media and the international attention to the topic, the control hospitals might have become more aware of their use of gloves, and improved. This shows that attention to the topic already has an effect by itself.

### Strengths and limitations

The strengths of this project are that we included a wide range of healthcare facilities, including acute care hospitals, tertiary-care centres, and LTCFs, and that we included two control hospitals outside of our region. This project also looked at proper glove use from different angles, including questionnaires, observations, procurement statistics, and interviews. These elements ensure the generalizability of the results of this project.

Limitations of our project include its execution during the COVID-19 pandemic, including the governmental regulations and restrictions that at times limited possibilities for glove observations because only staff essential for patient care was allowed. Connected to that, hospitals conducted before- and after measurements when they were ready, resulting in measurements in different seasons, which could have limited the effect of the intervention. Furthermore, the project was non-randomized, and the observers were not blinded. Next, there is a reduction in the number of participating healthcare facilities between the before and after measurements, which could have introduced selection bias in our project. Unfortunately, reasons for non-participation were not collected. Procurement statistics were difficult to obtain and were therefore available for only three healthcare facilities. Similar to studies in which hand hygiene is observed, the Hawthorne effect is present [32,33]. This is especially visible in the control hospitals, where also a positive effect was observed over time without having implemented the intervention. Finally, the COM-B questionnaire was not completed by all wards on which glove observations were performed, and the number of responses per ward differed.

This project shows the need for targeted interventions to address issues related to the opportunity to wear gloves correctly. While the percentage of incorrect usage was notably high, also after the intervention, awareness for this topic with a toolkit from which HCW could choose their (set of) tools appears to have a positive effect. Future studies should investigate the long-term effects of implementing the intervention, should study hand hygiene and glove compliance together, and study the effect on glove procurement statistics, and if different implementation strategies are needed for both hospitals and LTCFs.

## CRediT authorship contribution statement

**Anne F. Voor in ’t holt:** Writing – review & editing, Writing – original draft, Visualization, Methodology, Formal analysis, Data curation. **Robbert G. Bentvelsen:** Writing – review & editing, Methodology, Conceptualization. **Fleur Adriaanse:** Writing – review & editing, Project administration, Conceptualization. **Sabrina K. Been:** Writing – review & editing, Funding acquisition, Conceptualization. **Vicki Erasmus:** Writing – review & editing, Methodology, Conceptualization. **Juliëtte A. Severin:** Writing – review & editing, Supervision, Methodology, Conceptualization. **Jojanneke Dekkers:** Writing – review & editing, Investigation. **Tineke Heijerman:** Writing – review & editing, Investigation. **Yara Bamberg:** Writing – review & editing, Investigation. **Greet van der Tempel:** Writing – review & editing, Investigation. **Marianne van Groningen:** Writing – review & editing, Investigation. **Pia Gutierrez Zarate:** Writing – review & editing, Investigation. **Mieke Waltmans-den Breejen:** Writing – review & editing, Investigation. **Joy Kersten:** Writing – review & editing, Investigation. **Henriette Bakker:** Writing – review & editing, Investigation. **Bas de Jong:** Writing – review & editing, Investigation. **Wim van Vianen:** Writing – review & editing, Investigation. **Evelien van der Linden:** Writing – review & editing, Investigation. **Anja Bakker:** Writing – review & editing, Investigation.

## Ethical approval

Not required.

## Declaration of generative AI and AI-assisted technologies in the writing process

To improve the language and readability of this work, the authors used Microsoft Copilot. After using this tool, the authors reviewed and edited the content as needed and take full responsibility for the content of the publication.

## Funding sources

The ‘Goed Gebruik Handschoenen’ project was funded by the Infection Prevention and Antimicrobial Resistance Care Network South-Western Netherlands, Rotterdam, The Netherlands.

## Conflict of interest statement

The authors declare no conflicts of interest. M. W. co-developed the Hands in Control application together with Be-Remarkable. On our request, glove use was included, and this application was used in follow-up GGH initiatives. This application was not used for this project.

## Appendix A Supplementary data

Additional figure 1 Project timeline.

Additional file 1 The modified COM-B glove questionnaire (in Dutch).

Additional file 2 Methods of the Goed Gebruik Handschoenen (GGH) project

Additional file 3 Perceived usefulness interview questions.

Additional file 4 Results of the COM-B questionnaire.

Additional file 5 Glove procurement figures.

Additional file 6 Reasons for wearing gloves incorrectly.
