# Supplementary material for: Proper glove use: a multi-centre before–after improvement project
Source: Infect Prev Pract. 2026 Aug 7;8(4):100574. doi: 10.1016/j.infpip.2026.100574 (PMC13698051; doi:10.1016/j.infpip.2026.100574)
Supplement: Supplementary file 2 [file mmc2.docx]

**Vragenlijst handschoenen kennis**

Fijn dat je de tijd wilt nemen om deze vragenlijst in te vullen, dit duurt max. 5 minuten en is volledig anoniem. Deze vragenlijst is onderdeel van het project Goed Gebruik Handschoenen. Met dit project hopen wij inzicht te krijgen in het handschoenen gebruik van zorgprofessionals en jullie vervolgens handvatten te bieden voor bijvoorbeeld lastige situaties. Zo werken we samen aan een veilige zorgomgeving voor jou én de patiënt/cliënt/bewoner. Wil je de vragenlijst online invullen? Scan dan de QR-code met de camera van je telefoon en klik op de link die in beeld verschijnt.

In welke organisatie bent u werkzaam? ………………………………………………………

Op welke afdeling bent u werkzaam? ………………………………………………............

Wat is uw functie?...........................................................................................

Wat is uw leeftijd? (niet verplicht)………………………………………………………………

Lees elk item hieronder en omcirkel het nummer dat het beste aangeeft in hoeverre u het eens bent met elke stelling.

| 1. **Bekwaamheid** | Helemaal niet mee eens | Niet mee eens | Neutraal | Mee eens | Helemaal mee eens |
| --- | --- | --- | --- | --- | --- |
| a) Ik heb mijns inziens voldoende training over goed gebruik van handschoenen gehad (op deze afdeling) | 1 | 2 | 3 | 4 | 5 |
| b) Ik weet op welke momenten handschoenen dragen verplicht is | 1 | 2 | 3 | 4 | 5 |
| c) Ik weet op welke momenten je geen handschoenen hoort te dragen | 1 | 2 | 3 | 4 | 5 |
| d) Ik weet hoe ik op de juiste manier handschoenen moet dragen | 1 | 2 | 3 | 4 | 5 |
| e) Ik gebruik handschoenen zonder na te hoeven denken wanneer dit wel of niet noodzakelijk is | 1 | 2 | 3 | 4 | 5 |
| f) Ik vind het gemakkelijk om mij te houden aan de handschoenen richtlijnen (op deze afdeling) | 1 | 2 | 3 | 4 | 5 |
| 2. **Mogelijkheid** | Helemaal niet mee eens | Niet mee eens | Neutraal | Mee eens | Helemaal mee eens |
| a) Deze afdeling beschikt over voldoende en juiste handschoenen voorzieningen | 1 | 2 | 3 | 4 | 5 |
| b) Zorgprofessionals op deze afdeling dragen handschoenen altijd op de juiste momenten | 1 | 2 | 3 | 4 | 5 |
| c) Zorgprofessionals die deze afdeling bezoeken dragen handschoenen altijd op de juiste momenten | 1 | 2 | 3 | 4 | 5 |
| d) De handschoenen richtlijnen (van deze afdeling) zijn duidelijk | 1 | 2 | 3 | 4 | 5 |
| e) Er zijn geheugensteuntjes op deze afdeling om het personeel er aan te herinneren hoe en wanneer handschoenen gedragen moeten worden | 1 | 2 | 3 | 4 | 5 |
| 3. **Motivatie** | Helemaal niet mee eens | Niet mee eens | Neutraal | Mee eens | Helemaal mee eens |
| a) Naleving van handschoenen richtlijnen wordt als belangrijk beschouwd door personeel van deze afdeling | 1 | 2 | 3 | 4 | 5 |
| b) Naleving van handschoenen richtlijnen wordt als belangrijk beschouwd door mijn leidinggevende op deze afdeling | 1 | 2 | 3 | 4 | 5 |
| c) Ik streef naar volledige naleving van de handschoenen richtlijnen | 1 | 2 | 3 | 4 | 5 |
| d) Op deze afdeling attenderen wij elkaar op de juiste naleving van de handschoenen richtlijnen | 1 | 2 | 3 | 4 | 5 |
| e) Audits door de afdeling infectiepreventie stimuleren mij om mij te houden aan de handschoenen richtlijnen op deze afdeling | 1 | 2 | 3 | 4 | 5 |
| 4. **Gedrag** | Helemaal niet mee eens | Niet mee eens | Neutraal | Mee eens | Helemaal mee eens |
| **Ik draag altijd handschoenen:** |  |  |  |  |  |
| a) Bij (mogelijk) contact met bloed en andere lichaamsvloeistoffen | 1 | 2 | 3 | 4 | 5 |
| b) Bij (mogelijk) contact met slijmvliezen | 1 | 2 | 3 | 4 | 5 |
| c) Bij (mogelijk) contact met niet-intacte huid | 1 | 2 | 3 | 4 | 5 |
| d) Bij contact met verpleeg- en behandelmaterialen dat in contact is geweest met bloed, lichaamsvloeistof, slijmvliezen of niet-intacte huid | 1 | 2 | 3 | 4 | 5 |
| e) Wanneer ik een beschadiging aan de huid op mijn hand(en) heb | 1 | 2 | 3 | 4 | 5 |
| f) Wanneer ik werk met een patiënt/cliënt die een isolatie indicatie heeft | 1 | 2 | 3 | 4 | 5 |
| g) Wanneer ik werk met medicijnen en stoffen die via de huid kunnen worden opgenomen | 1 | 2 | 3 | 4 | 5 |
| h) Wanneer ik werk met irriterende of bijtende stoffen | 1 | 2 | 3 | 4 | 5 |
| i) Wanneer ik materialen of oppervlakten desinfecteer | 1 | 2 | 3 | 4 | 5 |
| j) Die te groot of te klein zijn | 1 | 2 | 3 | 4 | 5 |
| k) Met sieraden, nagellak, kunstnagels of een brace eronder | 1 | 2 | 3 | 4 | 5 |
| l) Met een tweede paar daar overheen | 1 | 2 | 3 | 4 | 5 |
| m) En desinfecteer of was deze | 1 | 2 | 3 | 4 | 5 |
| n) En kom daarmee in contact met het eigen gezicht, persoonlijke beschermingsmiddelen en de omgeving of omgevingsmaterialen | 1 | 2 | 3 | 4 | 5 |
| o) En loop daarmee in de openbare ruimte (de gang, de lift, etc.) | 1 | 2 | 3 | 4 | 5 |
| **Ik verwissel altijd handschoenen:** | 1 | 2 | 3 | 4 | 5 |
| p) Tussen het verzorgen van verschillende patiënten/cliënten | 1 | 2 | 3 | 4 | 5 |
| q) Bij handelingen van vuil naar schoon | 1 | 2 | 3 | 4 | 5 |
| r) Wanneer ze kapot of zichtbaar verontreinigd zijn | 1 | 2 | 3 | 4 | 5 |

Mocht je vragen hebben, mail naar [abr.zwn@erasmusmc.nl](mailto:abr.zwn@erasmusmc.nl). Heel erg bedankt voor je medewerking!
