# Supplementary material for: Proper glove use: a multi-centre before–after improvement project
Source: Infect Prev Pract. 2026 Aug 7;8(4):100574. doi: 10.1016/j.infpip.2026.100574 (PMC13698051; doi:10.1016/j.infpip.2026.100574)
Supplement: Supplementary file 3 [file mmc3.docx]

**Additional file 2.** Methods of the GGH project

**The questionnaire**

To gain insight into the knowledge of HCW regarding the use of gloves, the existing COM-B questionnaire that had been used to study hand hygiene was adapted to focus on glove use. The questionnaire centres on four themes: Capability, Opportunity, Motivation, and Behaviour.^[1],^^[2]^ The questionnaire was translated from English into Dutch by van Dijk et al.; the GGH project team replaced all references to hand hygiene by gloves, and the theme Behaviour was adjusted to reflect the developed regional policy (see Additional file 1). We created a digital version of the questionnaire using an online form and a paper version that could be printed by the local project leaders, allowing them to distribute it on paper within their organizations. Furthermore, we created a manual which included suggestions on how the local project leaders could best distribute the questionnaire. Answers provided via the Google Form, on paper (send via mail or a scanned version via e-mail) were collected in a Microsoft Excel database. Answers were grouped by department. If only one or two HCW of a department filled out the questionnaire, they were grouped as ‘other’ as their anonymity could not be guaranteed. For each of the four themes, a maximum number of 'points' could be scored. If this maximum was 30 and the questionnaire was completed by 17 HCW, the maximum score for that theme would be 510 points. The given answers were summed, for example, 410 in total. This was then calculated as 410/510 x 100% = 80.4%. This percentage represented the score for that theme for a specific department. The same method was applied for each theme and department, and the results were presented in a table within a report. A higher percentage indicated higher agreement with the statements by the HCW. After sharing the report with the local project leaders, the GGH project team advised on tailored interventions.

**The toolkit**

Based on the input received during the GGH kick-off meeting and in cooperation with the local project leaders, tools for the toolkit were designed. It was decided to gather bottom-up input from the participants to ensure the tools aligned as closely as possible with the needs of the healthcare facilities. Following this input, a first version of the toolkit was developed by the IPCN-SWN in cooperation with a communication advisor, and reviewed by the GGH project advisory board. Local project leaders could give feedback on this first version, and tools were adjusted accordingly. The final developed box was sent to participating centres with all printable materials in it (Table 1) starting from November 16, 2021. All available tools were published on the project website.^[3]^ In March 2022, the toolkit was evaluated in the local project leaders meeting. During this meeting, it became clear that an additional poster was needed, with pictures showing how to safely put on and take off gloves. Additionally, it was expressed that it would be beneficial to develop stickers with reminders of proper glove use. Both items were subsequently developed and included in the toolkit and made available online.

**Table 1.** Overview of the toolkit tools

| **Tool** | **Description** |
| --- | --- |
| Facts and myths | A document was designed to discuss the correct use of gloves by presenting colleagues with statements where they must decide whether each one is a fact or a myth. |
| Videos | Videos were created for hospitals and LTCFs, first showing a scenario where gloves are used incorrectly, illustrating the spread of bacteria. The correct method is then demonstrated, followed by a summary of proper and improper glove use. |
| Icons | Various icons were developed for use in presentations or other written materials. |
| Game | A game was developed where HCW could answer questions about proper glove use in a playful way. The game features various levels and is designed to be played as quickly as possible to achieve the highest score. |
| Image library | Photos taken for the posters were included in an image library, allowing participants to download and use them for presentations and training sessions. |
| Posters | Posters were created with images illustrating correct and incorrect (improper) glove use. Furthermore, a poster was developed to demonstrate how to properly put on and take off gloves. |
| Stickers | Stickers with various reminders were designed to be placed on glove boxes. The quotes would help remind staff when to wear or not wear gloves and emphasize that hand hygiene must always be performed before donning and after doffing gloves. |

Abbreviations: HCW; healthcare worker, LTCFs; long-term care facilities.

Participating healthcare facilities were allowed to select a subset of the available tools to tailor the intervention to the local needs. A manual on how to use the tools was written together with a communication advisor. Additionally, a document with general tips for each organization on how to communicate and report the results of the observations and project toolkit tools (Table 1) within the organization was designed and provided.

**Observations**

Local project leaders had the option to only chose tools from the toolkit or to also measure proper glove use via observation tools (Table 1). This was not mandatory, but strongly recommended. When performing measurements, it was advised to first do a baseline-before measurement, followed by implementing the toolkit, and finally a second-after measurement. It was also possible to only do a baseline measurement without further follow-up measurements. A manual on how to perform the observations on proper glove use was created together with a communication advisor and send to the local project leaders. A Google Form was developed for the glove observations. In this Google Form the following questions were asked: 1) In which healthcare facility are you observing? 2) At which department are you observing? 3) Where are you located at the department? 4) Which HCW are you observing? 5) Does the HCW need to wear gloves according to the regional policy? 6) Is the HCW wearing gloves? 7) If yes to 6, are the gloves being worn correctly? 8) If no to 7, what is going wrong? The manual provided detailed instructions on our recommendations for conducting the observations. For example: 1) observe for two half-days, 2) follow a nurse or stand in a fixed spot and observe passers-by, 3) do not specifically look for HCW wearing gloves; instead, observe the HCW caring for patients and whether gloves are being worn or not. For each situation/action, a new Google Form had to be filled out. It was advised that either the local project leader, an IPC practitioner, a HCW specialized in infection prevention, or a HCW familiar with the GGH project would perform the observations. During a local project leaders meeting, the manual was introduced, explained, and discussed. This was based on the teach-the-teacher principle, in which the GGH project team informed the local project leaders, and the local project leaders informed the HCW performing the observations in their facility. This meeting was recorded allowing participants who were unable to attend to watch it at a later time. It was recommended to collect data from the same departments when conducting before-and-after measurements to facilitate analysis and reporting.

After the ‘before’ measurements were completed, the GGH project team created reports per observed department. It was advised to communicate the results with the respective department and to choose and implement tools from the toolkit together. For departments also performing ‘after’ measurements, a final report was created showing the differences. Departments from two hospitals outside of the IPCN-SWN were asked to be the control group. They did not have access to the toolkit and only did two rounds of observations with some months in between.

1. Lydon S, Greally C, Tujjar O, Reddy K, Lambe K, Madden C, et al. Psychometric evaluation of a measure of factors influencing hand hygiene behaviour to inform intervention. J Hosp Infect 2019;102(4):407-1.
2. van Dijk MD, Nieboer D, Vos MC, van Beeck EF. Validity of self-reported compliance and behavioural determinants of observed compliance: an application of the COM-B hand hygiene questionnaire in nine Dutch hospitals. J Hosp Infect 2023;137:61-8.
3. IP & AMR zorgnetwerk Zuidwest-Nederland. Toolkit, <https://amrzorgnetwerkzwn.nl/verpleeghuiszorg/goed-gebruik-handschoenen/toolkit/>; 2025.
