# Supplementary material for: Proper glove use: a multi-centre before–after improvement project
Source: Infect Prev Pract. 2026 Aug 7;8(4):100574. doi: 10.1016/j.infpip.2026.100574 (PMC13698051; doi:10.1016/j.infpip.2026.100574)
Supplement: Supplementary file 4 [file mmc4.docx]

**Additional file 3.** Perceived usefulness interview questions

**Date:**

**Healthcare centre:**

**Local project leader:**

1. Who (healthcare worker/roles) were involved in this project?
2. Which tools from the “Goed Gebruik Handschoenen” (GGH) toolkit or other interventions did you implement and use to promote the proper use of gloves? [These could be project interventions, but also ask openly about any other actions taken during this period.]
   a. Facts and myths
   b. Videos
   c. Icons
   d. Game
   e. Image library
   f. Posters
   g. Stickers
   h. Did you perform glove observations using the observation tools?
   i. Did you implement the regional glove policy?
3. How did you use the GGH toolkit and other interventions? [For each component, ask for a brief description of how it was used. Consider:
   a. Who used it
   b. How it reached them [training/email, etc.]
   c. Voluntary or mandatory participation
   d. Uptake, in other words, how much it was actually used
   e. What were the reactions to that component
4. Which parts of the toolbox did you find most useful? Why? Can you provide an example of how it was used? Is this the experience/opinion of the local project leader or is this feedback from the healthcare workers (HCW)/healthcare centre?
5. Which parts of the toolbox were considered the least useful? Can you explain why? Is this the experience/opinion of the local project leader or is this feedback from the HCW/healthcare centre?
6. What did you feel was missing from the toolkit? In other words, what improvements could be made to the GGH toolkit?
7. What rating would you give the GGH toolkit? (1 = very negative, 10 = very positive) 1 2 3 4 5 6 7 8 9 10; Can you explain why? Is this the experience/opinion of the local project leader or is this feedback from the HCW/healthcare centre?
8. Were there any unexpected developments during this improvement process? [Consider other factors within the organization, etc.] How could we prevent this in the future?
