# Supplementary material for: Proper glove use: a multi-centre before–after improvement project
Source: Infect Prev Pract. 2026 Aug 7;8(4):100574. doi: 10.1016/j.infpip.2026.100574 (PMC13698051; doi:10.1016/j.infpip.2026.100574)
Supplement: Supplementary file 5 [file mmc5.docx]

**Additional file 4.** Results of the modified COM-B questionnaire.

**Table 1.** Modified COM-B questionnaire results (n= 1,111 healthcare workers).

| **Theme** | **Number of questions** | **Maximum number of points** | **Median (range)** | **Median % (range)** |
| --- | --- | --- | --- | --- |
| Capability | 6 | 30 | 24 (7-30) | 80% (23.3%-100%) |
| Opportunity | 5 | 25 | 18 (7-25) | 72% (28%-100%) |
| Motivation | 5 | 25 | 19 (7-25) | 76% (28%-100%) |
| Behaviour | 18 | 90 | 81 (43-90) | 90% (47.8%-100%) |

**Figure 1.** Results of the modified COM-B questionnaire, n=1111 healthcare workers.

**Figure 2.** COM-B results for hospitals (n=8, n=819 healthcare workers).

**Figure 3.** COM-B results for long-term care facilities (n=5, n=292 healthcare workers).

**Figure 4a-d.** COM-B results for the top 5 healthcare worker functions: nurse (n=583), practical nurse (n=132), nurse in training (n=62), laboratory technician (n=57), and nursing assistant (n=48); also displaying the results of HCW with another function (n=229).

a. **Capability**

b. **Opportunity**

c. **Motivation**

d. **Behaviour**
