# Supplementary material for: Proper glove use: a multi-centre before–after improvement project
Source: Infect Prev Pract. 2026 Aug 7;8(4):100574. doi: 10.1016/j.infpip.2026.100574 (PMC13698051; doi:10.1016/j.infpip.2026.100574)
Supplement: Supplementary file 6 [file mmc6.docx]

**Additional file 5.** Glove procurement figures

Glove units

|  | Hospital 1 | LTCF 1 | Hospital 2 | Hospital 3 | Hospital 4 | LTCF 2 |
| --- | --- | --- | --- | --- | --- | --- |
| 2019 January |  |  | 138600 | 78410 |  | 35230 |
| 2019 February |  |  | 146500 | 68200 |  | 20210 |
| 2019 March |  |  | 137700 | 76660 |  | 17700 |
| 2019 April |  |  | 133200 | 81190 |  | 16500 |
| 2019 May |  |  | 137500 | 76694 |  | 30000 |
| 2019 June |  |  | 139900 | 69740 |  | 24450 |
| 2019 July |  |  | 153000 | 73080 |  | 36600 |
| 2019 August |  |  | 127900 | 53080 |  | 18950 |
| 2019 September |  |  | 156500 | 64350 |  | 27600 |
| 2019 October |  |  | 156900 | 73650 |  | 23250 |
| 2019 November |  |  | 171100 | 68400 |  | 29100 |
| 2019 December |  |  | 174300 | 78400 |  | 35550 |
| 2020 January | 148474 | 65235 | 172400 | 93970 |  | 18850 |
| 2020 February | 108410 | 105373 | 159000 | 69004 |  | 21900 |
| 2020 March | 63943 | 23226 | 216700 | 47660 |  | 19350 |
| 2020 April | 11491 | 535 | 85700 | 2500 |  | 25650 |
| 2020 May | 8736 | 15727 | 93200 | 1000 |  | 41250 |
| 2020 June | 7534 | 8193 | 83600 | 0 |  | 20200 |
| 2020 July | 10886 | 12927 | 83300 | 500 |  | 39600 |
| 2020 August | 14820 | 3104 | 135500 | 24 |  | 9900 |
| 2020 September | 13945 | 2445 | 140700 | 0 |  | 3000 |
| 2020 October | 10609 | 2088 | 205500 | 0 |  | 1650 |
| 2020 November | 8381 | 3893 | 175300 | 36 |  | 11150 |
| 2020 December | 9683 | 4399 | 218500 | 24 |  | 41500 |
| 2021 January | 10817 | 1498 | 157700 | 0 | 204 | 26700 |
| 2021 February | 10030 | 1307 | 164100 | 0 | 205 | 450 |
| 2021 March | 9857 | 1981 | 200500 | 12 | 217 | 2250 |
| 2021 April | 11439 | 1584 | 114200 | 48 | 262 | 900 |
| 2021 May | 7967 | 1114 | 104200 | 0 | 325 | 900 |
| 2021 June | 9568 | 1451 | 131300 | 21210 | 257 | 19200 |
| 2021 July | 8333 | 895 | 111600 | 54350 | 245 | 99150 |
| 2021 August | 6610 | 1179 | 115500 | 61196 | 257 | 1200 |
| 2021 September | 7512 | 785 | 94300 | 76080 | 288 | 6750 |
| 2021 October | 7325 | 842 | 105600 | 59400 | 344 | 30450 |
| 2021 November | 7099 | 1048 | 123000 | 84490 | 490 | 56100 |
| 2021 December | 6372 | 873 | 131700 | 58422 | 319 | 40800 |
| 2022 January | 6928 | 722 | 102800 | 57540 | 260 | 16800 |
| 2022 February | 7270 | 755 | 114100 | 65868 | 248 | 35100 |
| 2022 March | 7465 | 641 | 107500 | 75540 | 274 | 41100 |
| 2022 April | 6417 | 731 | 120500 | 77260 | 288 | 23100 |
| 2022 May | 6448 | 695 | 118400 | 60270 | 348 | 29550 |
| 2022 June | 7375 | 839 | 147100 | 64950 | 261 | 37350 |
| 2022 July | 7320 | 558 | 126800 | 66960 | 321 | 50550 |
| 2022 August | 5795 | 780 | 108700 | 68832 | 298 | 16500 |
| 2022 September | 7827 | 860 | 134200 | 68722 | 241 | 45900 |
| 2022 October | 6581 | 881 | 129600 | 69564 | 321 | 29250 |
| 2022 November | 7572 | 725 | 130900 | 79714 | 326 | 35550 |
| 2022 December | 6536 | 781 | 139000 | 82474 | 253 |  |
| 2023 January | 7603 | 833 |  | 77240 | 266 |  |
| 2023 February | 5839 | 761 |  | 77942 | 282 |  |
| 2023 March | 7896 | 833 |  | 79762 | 249 |  |
| 2023 April | 6703 | 1088 |  | 55634 | 251 |  |
| 2023 May | 6552 | 827 |  | 74472 | 280 |  |
| 2023 June | 7561 | 815 |  | 67060 | 253 |  |
| 2023 July | 4743 | 411 |  |  | 180 |  |

**Figure 1.** Glove procurement statistics for 6 healthcare centres. Due to the unstable figures during the COVID-19 pandemic, data from 2019 was compared to 2022 (blue vertical lines). The units on the y-axis were expressed differently in each healthcare centre. Abbreviations: LTFC; long-term care facility.

**Hospital 2**

- Mean number of glove units 2019 = 147758.33
- Mean number of glove units 2022 = 123300.00

P-value <0.001

**Hospital 3**

- Mean number of glove units 2019 = 71821.17
- Mean number of glove units 2022 = 69807.83

P-value 0.528

**LTCF 2**

- Mean number of glove units 2019 = 26261.67
- Mean number of glove units 2022 = 32795.45

P-value 0.105
