# Supplementary material for: Proper glove use: a multi-centre before–after improvement project
Source: Infect Prev Pract. 2026 Aug 7;8(4):100574. doi: 10.1016/j.infpip.2026.100574 (PMC13698051; doi:10.1016/j.infpip.2026.100574)
Supplement: Supplementary file 7 [file mmc7.docx]

**Additional file 6.** Reasons for wearing gloves incorrectly

**Table 1.** Reasons for incorrectly wearing gloves (n=671) in the *before* period of the included hospitals (n=10).

| **Reason for incorrectly wearing gloves** | **No. reported** | **%** |
| --- | --- | --- |
| (1) No hand hygiene before putting on gloves | 175 | 26.1 |
| (2) No hand hygiene after taking off gloves | 85 | 12.7 |
| (3) Touches, while having gloves on, the environment (e.g., phone, doorknob, keyboard) or the patient environment | 74 | 11.0 |
| (4) Does not change gloves when working from dirty to clean environments | 49 | 7.3 |
| (5) Wears gloves in public spaces (e.g., the hallway, the elevator) | 49 | 7.3 |
| (3) and (5) | 29 | 4.3 |
| (1) and (2) | 24 | 3.6 |
| (1) and (3) | 24 | 3.6 |
| (1) and (2) and (3) | 12 | 1.8 |
| (2) and (3) | 12 | 1.8 |
| (6) Disinfects gloves or washes hands with gloves on | 10 | 1.5 |
| (1) and (2) and (3) and (5) | 10 | 1.5 |
| (7) Touches the face with gloves | 9 | 1.3 |
| (8) Wears gloves which are either too big or too small | 8 | 1.2 |
| (9) Wears multiple gloves | 6 | 0.9 |
| (2) and (3) and (5) | 6 | 0.9 |
| (1) and (2) and (3) and (4) | 5 | 0.7 |
| (3) and (4) | 5 | 0.7 |
| (4) and (5) | 4 | 0.6 |
| (10) Wears jewelry, nail polish, artificial nails, or a brace under the gloves | 4 | 0.6 |
| (11) Uses the same gloves for multiple patients | 4 | 0.6 |
| (1) and (4) | 3 | 0.4 |
| (1) and (3) and (4) | 3 | 0.4 |
| (1) and (5) | 3 | 0.4 |
| (2) and (6) | 3 | 0.4 |
| (2) and (4) | 2 | 0.3 |
| (1) and (3) and (4) and (5) | 2 | 0.3 |
| (2) and (5) | 2 | 0.3 |
| (1) and (2) and (3) and (4) and (5) | 2 | 0.3 |
| (1) and (3) and (5) | 2 | 0.3 |
| (12) Touches personal protective equipment with gloves | 2 | 0.3 |
| (6) and (11) | 2 | 0.3 |
| (3) and (11) | 2 | 0.3 |
| (1) and (12) | 1 | 0.1 |
| (3) and (5) and (11) | 1 | 0.1 |
| (3) and (12) | 1 | 0.1 |
| (2) and (3) and (4) and (5) and (7) | 1 | 0.1 |
| (6) and (10) | 1 | 0.1 |
| (5) and (10) | 1 | 0.1 |
| (1) and (11) | 1 | 0.1 |
| (1) and (2) and (3) and (5) and (11) | 1 | 0.1 |
| (1) and (3) and (8) | 1 | 0.1 |
| (1) and (2) and (8) | 1 | 0.1 |
| (13) Wears dirty or damaged gloves | 1 | 0.1 |
| (1) and (2) and (3) and (5) and (7) and (8) | 1 | 0.1 |
| (14) Replaces one glove without performing hand hygiene | 1 | 0.1 |
| (1) and (2) and (5) | 1 | 0.1 |
| (1) and (2) and (3) and (5) and (10) | 1 | 0.1 |
| (2) and (3) and (12) | 1 | 0.1 |
| (1) and (2) and (4) | 1 | 0.1 |
| (1) and (4) and (11) | 1 | 0.1 |
| (5) and (11) | 1 | 0.1 |
| (2) and (15) makes the bed with gloves on | 1 | 0.1 |
| (2) and (3) and (4) and (5) | 1 | 0.1 |
| (1) and (3) and (7) | 1 | 0.1 |
| (2) and (3) and (11) | 1 | 0.1 |
| (1) and (5) and (12) | 1 | 0.1 |
| (2) and (3) and (5) and (7) and (11) and (12) | 1 | 0.1 |
| (2) and (3) and (8) | 1 | 0.1 |
| (3) and (4) and (5) and (8) | 1 | 0.1 |
| (16) Performs hand hygiene incorrectly, with 70% alcohol instead of hand alcohol | 1 | 0.1 |
| (2) and (17) combs the hair of a patient with a glove | 1 | 0.1 |
| (1) and (4) and (6) | 1 | 0.1 |
| (5) and (7) | 1 | 0.1 |
| (1) and (4) and (5) | 1 | 0.1 |
| (1) and (3) and (4) and (11) | 1 | 0.1 |
| (2) and (18) takes off gloves and puts it in the pocket of the uniform | 1 | 0.1 |
| (1) and (3) and (5) and (11) | 1 | 0.1 |
| (1) and (8) | 1 | 0.1 |
| (3) and (5) and (8) | 1 | 0.1 |
| (2) and (3) and (4) | 1 | 0.1 |
| (1) and (3) and (5) and (10) | 1 | 0.1 |

Abbreviations: no; number.

**Table 2.** Reasons for incorrectly wearing gloves (n=319) in the *after* period of the included hospitals (n=5).

| **Reason for incorrectly wearing gloves** | **No. reported** | **%** |
| --- | --- | --- |
| (1) No hand hygiene before putting on gloves | 102 | 32.0 |
| (2) No hand hygiene after taking off gloves | 54 | 16.9 |
| (1) and (3) | 29 | 9.1 |
| (3) Touches, while having gloves on, the environment (e.g., phone, doorknob, keyboard) or the patient environment | 28 | 8.8 |
| (4) Does not change gloves when working from dirty to clean environments | 19 | 6.0 |
| (1) and (2) | 13 | 4.1 |
| (1) and (3) and (6) | 8 | 2.5 |
| (5) Wears multiple gloves | 5 | 1.6 |
| (6) Wears gloves in public spaces (e.g., the hallway, the elevator) | 5 | 1.6 |
| (1) and (2) and (3) | 5 | 1.6 |
| (1) and (2) and (3) and (6) | 5 | 1.6 |
| (2) and (3) | 4 | 1.3 |
| (7) Touches the face with gloves | 4 | 1.3 |
| (1) and (6) | 4 | 1.3 |
| (1) and (3) and (4) | 4 | 1.3 |
| (8) Uses the same gloves for multiple patients | 3 | 0.9 |
| (3) and (8) | 3 | 0.9 |
| (3) and (4) and (8) | 2 | 0.6 |
| (1) and (2) and (6) | 2 | 0.6 |
| (9) The glove was under the cuff of the isolation gown | 1 | 0.3 |
| (10) Touches personal protective equipment with gloves | 1 | 0.3 |
| (1) and (6) and (10) | 1 | 0.3 |
| (3) and (4) | 1 | 0.3 |
| (2) and (3) and (4) and (10) | 1 | 0.3 |
| (1) and (2) and (3) and (8) | 1 | 0.3 |
| (11) Disinfects gloves or washes hands with gloves on | 1 | 0.3 |
| (1) and (2) and (8) | 1 | 0.3 |
| (2) and (3) and (10) | 1 | 0.3 |
| (12) Wears jewelry, nail polish, artificial nails, or a brace under the gloves | 1 | 0.3 |
| (2) and (3) and (4) and (6) and (10) | 1 | 0.3 |
| (1) and (2) and (10) | 1 | 0.3 |
| (1) and (3) and (4) and (6) | 1 | 0.3 |
| (1) and (2) and (4) | 1 | 0.3 |
| (1) and (2) and (3) and (4) | 1 | 0.3 |
| (1) and (5) | 1 | 0.3 |
| (1) and (4) | 1 | 0.3 |
| (1) and (3) and (8) | 1 | 0.3 |
| (1) and (2) and (3) and (10) | 1 | 0.3 |
| (1) and (8) | 1 | 0.3 |

Abbreviations: no; number.

**Table 3.** Reasons for incorrectly wearing gloves (n=117) in the *before* period of the control hospitals (n=2).

| **Reason for incorrectly wearing gloves** | **No. reported** | **%** |
| --- | --- | --- |
| (1) Touches, while having gloves on, the environment (e.g., phone, doorknob, keyboard) and/or the patient environment | 42 | 35.9 |
| (2) No hand hygiene after taking off gloves | 19 | 16.2 |
| (3) Wears gloves in public spaces (e.g., the hallway, the elevator) | 10 | 8.5 |
| (4) No hand hygiene before putting on gloves | 6 | 5.1 |
| (5) Does not change gloves when working from dirty to clean environments | 4 | 3.4 |
| (1) and (2) and (4) | 4 | 3.4 |
| (1) and (4) | 4 | 3.4 |
| (1) and (6) | 3 | 2.6 |
| (1) and (5) | 3 | 2.6 |
| (6) Uses the same gloves for multiple patients | 2 | 1.7 |
| (2) and (4) | 2 | 1.7 |
| (7) Wears dirty gloves multiple times | 2 | 1.7 |
| (3) and (8) | 2 | 1.7 |
| (8) Wears gloves while cleaning and gloves are not necessary (i.e., mopping the floor) | 2 | 1.7 |
| (2) and (3) | 1 | 0.9 |
| (2) and answers the phone without gloves and without hand hygiene | 1 | 0.9 |
| (1) and (2) and (9) | 1 | 0.9 |
| (3) and (4) | 1 | 0.9 |
| (1) and (2) and (4) and (6) and (9) and (10) | 1 | 0.9 |
| (1) and (2) and (4) and (9) | 1 | 0.9 |
| (1) and (3) and (4) | 1 | 0.9 |
| (1) and rinses the gloves under the tap/running water | 1 | 0.9 |
| (9) Touches the face with gloves | 1 | 0.9 |
| (9) and (10) | 1 | 0.9 |
| (10) Touches personal protective equipment with gloves | 1 | 0.9 |
| (10) and is touching work clothing with a gloved hand | 1 | 0.9 |

Abbreviations: no; number.

**Table 4.** Reasons for incorrectly wearing gloves (n=93) in the *after* period of the control hospitals (n=2).

| **Reasons for incorrectly wearing gloves** | **No. reported** | **%** |
| --- | --- | --- |
| (1) Touches, while having gloves on, the environment (e.g., phone, doorknob, keyboard) and/or the patient environment | 29 | 31.2 |
| (3) Wears gloves in public spaces (e.g., the hallway, the elevator) | 10 | 10.8 |
| (2) No hand hygiene after taking off gloves | 10 | 10.8 |
| (5) Does not change gloves when working from dirty to clean environments | 10 | 10.8 |
| (2) and (4) | 8 | 8.6 |
| (4) No hand hygiene before putting on gloves | 7 | 7.5 |
| (1) and (2) and (4) | 4 | 4.3 |
| (9) Touches the face with gloves | 2 | 2.2 |
| (6) Uses the same gloves for multiple patients | 1 | 1.1 |
| Continues wearing gloves while they are damaged or visibly contaminated | 1 | 1.1 |
| (3) and wears two gloves on one hand | 1 | 1.1 |
| (8) Wears gloves while cleaning and gloves are not necessary (i.e., mopping the floor) | 1 | 1.1 |
| (2) and (3) | 1 | 1.1 |
| (1) and (4) | 1 | 1.1 |
| There is no need to wear gloves for these tasks | 1 | 1.1 |
| Does not change the gloves frequently | 1 | 1.1 |
| Touches the medication cart in the hallway with gloved hands | 1 | 1.1 |
| (1) and (3) and does not change the gloves in between the two | 1 | 1.1 |
| (1) and there is no need to wear gloves for these tasks | 1 | 1.1 |
| Has gloved hands in the cupboards after contact with the patient and/or patient environment | 1 | 1.1 |
| Unknown | 1 | 1.1 |

Abbreviations: no; number.
