# Supplementary material for: Proper glove use: a multi-centre before–after improvement project
Source: Infect Prev Pract. 2026 Aug 7;8(4):100574. doi: 10.1016/j.infpip.2026.100574 (PMC13698051; doi:10.1016/j.infpip.2026.100574)
Supplement: Supplementary file 8 [file mmc1.docx]

**Additional figure 1.** Project timeline
